# Eosinophilic Esophagitis Revealed by Hematemesis in an Asthmatic Adolescent: A Case Report

**DOI:** 10.7759/cureus.115035

**Published:** 2026-08-23

**Authors:** Karima El Fakiri, Chaima Haidar, Aicha Bourrahouat, Noureddine Rada, Mohammed Bouskraoui

**Affiliations:** 1 Department of Pediatrics A, Mohammed VI University Hospital, Faculty of Medicine and Pharmacy, Cadi Ayyad University, Marrakech, MAR; 2 Department of Pediatrics B, Mohammed VI University Hospital, Faculty of Medicine and Pharmacy, Cadi Ayyad University, Marrakech, MAR

**Keywords:** adolescent, asthma, eosinophilic esophagitis, hematemesis, topical corticosteroids

## Abstract

Eosinophilic esophagitis (EoE) is a chronic inflammatory condition of the esophagus, with increasing prevalence and incidence, characterized by eosinophilic infiltration of the mucosa, and frequently associated with asthma and atopic predisposition.

We report the case of a 13-year-old female patient with poorly controlled moderate persistent asthma, admitted for recurrent vomiting over the past year, complicated by the sudden onset of hematemesis, associated with fever and clinical deterioration. On admission, the patient presented with tachycardia, hypoxemia, and acute respiratory distress associated with an asthma exacerbation.

An emergency esophagogastroduodenoscopy revealed EoE, with histopathology demonstrating dense eosinophilic infiltration (>15 eosinophils/high-power field). Allergy testing revealed food sensitization to prawns. Treatment with oral fluticasone (250 μg twice daily) combined with double-dose proton pump inhibitors resulted in rapid clinical remission and complete histological normalization at 10 months.

This case highlights the need to systematically consider EoE in the presence of any acute gastrointestinal symptoms in a patient with atopic predisposition, and the importance of early management.

## Introduction

Eosinophilic esophagitis (EoE) is a chronic inflammatory disease of the esophagus characterized by symptoms of esophageal dysfunction and a predominant eosinophilic infiltration of the mucosa. It results from a Th2-polarized immune response to food or environmental antigens in genetically predisposed individuals, in which cytokines such as IL-5, IL-13, and eotaxin-3 drive eosinophil recruitment and activation in the esophageal mucosa [1].

Its prevalence and incidence are steadily increasing: a 2023 global systematic review and meta-analysis estimated a pooled incidence of 5.31 cases per 100,000 inhabitant-years and a pooled prevalence of 40.04 cases per 100,000 inhabitant-years [2].

EoE is frequently associated with atopic conditions, particularly asthma, with asthma reported in 30% to 50% of affected children [3,4]. Clinical manifestations in children are heterogeneous and vary according to age, ranging from feeding difficulties and vomiting in young children to dysphagia and food impaction in adolescents [5]. Diagnosis is based on a combination of clinical, endoscopic, and histological findings; esophagogastroduodenoscopy (EGD) with multiple biopsies remains essential when the condition is suspected [1,6]. Upper gastrointestinal bleeding, particularly hematemesis, remains a distinctly atypical presentation of EoE, with only a few pediatric cases reported to date [7,8].

We report the case of a 13-year-old patient with poorly controlled persistent asthma in whom atypical digestive symptoms, notably hematemesis, led to the diagnosis of EoE, an unusual and alarming presentation that underscores the importance of considering EoE in the differential diagnosis of acute upper gastrointestinal bleeding in atopic children, even in the absence of the more classic symptom of dysphagia.

## Case presentation

A 13-year-old female patient with a history of *Helicobacter pylori* gastritis, diagnosed 18 months earlier by gastric biopsy with a rapid urease test and treated with standard triple therapy for eradication, as well as persistent, poorly controlled moderate asthma, was admitted for recurrent episodes of cyclic vomiting over a period of one year. The episodes had progressively worsened over the two weeks preceding admission, complicated by the sudden onset of moderate hematemesis; the volume was not formally quantified, accompanied by fever, asthenia, and weight loss. No melena was reported. She was receiving maintenance therapy with inhaled fluticasone combined with a long-acting β2-agonist (salmeterol), along with salbutamol as needed, and had poor treatment adherence.

Upon admission, the patient was conscious but presented with tachycardia, hypoxemia, and acute respiratory distress. Oxygen saturation was 85% on room air, heart rate was 112 bpm, respiratory rate was 40 breaths per minute, blood pressure was 114/74 mmHg, and temperature was 38.7°C. Physical examination revealed pallor of the skin and mucous membranes. Her weight was 40 kg (-1 standard deviation), her height was 155 cm, and her urine output was 1.8 mL/kg/hour.

Chest auscultation revealed bilateral wheezing and crackles, suggestive of an asthma exacerbation. Abdominal examination was unremarkable, with no organomegaly or signs of portal hypertension.

Initial management included salbutamol nebulization, paracetamol, octreotide (Sandostatin) for 48 hours, and high-dose proton pump inhibitors, without improvement in gastrointestinal symptoms. No requirement for blood transfusion.

Laboratory investigations showed hemoglobin of 13.3 g/dL, platelet count of 286,000/mm³, eosinophil count of 37/mm³, prothrombin time of 80.5%, and activated partial thromboplastin time of 25 seconds.

**Figure 1:**
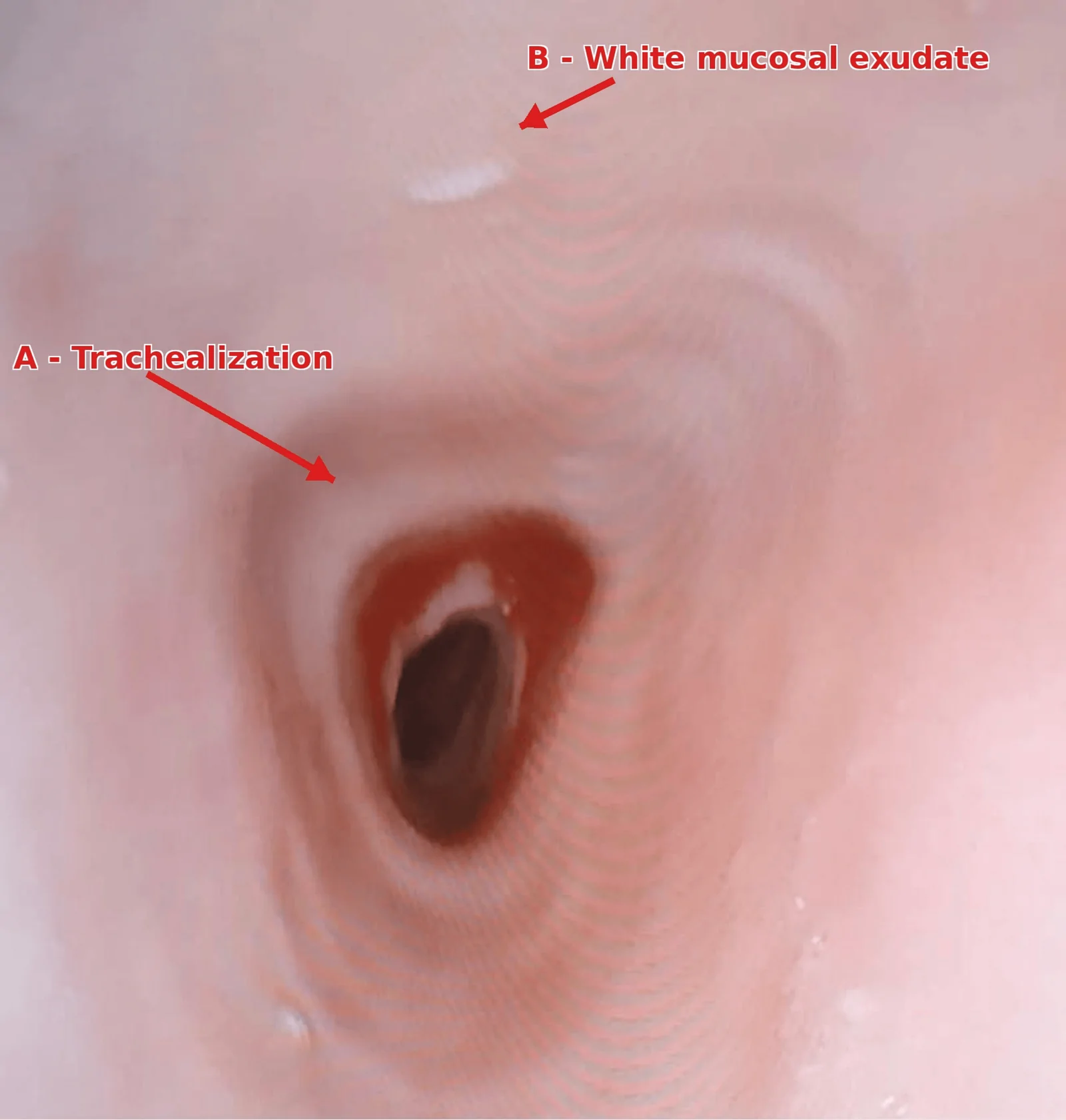
Endoscopic findings in eosinophilic esophagitis; esophageal trachealization and white exudates.

An emergency EGD was performed, revealing endoscopic features suggestive of EoE, including esophageal trachealization with concentric rings and white mucosal exudates (EREFS: rings and exudates present; mild diffuse edema also noted, without furrows or strictures) (Figure 1).

Multiple biopsies were obtained (six in total, two each from the proximal, middle, and distal esophagus). Histological examination demonstrated marked eosinophilic infiltration exceeding 15 eosinophils per high-power field (HPF), with evidence of eosinophilic microabscesses. Gastric biopsies were normal, with no evidence of *Helicobacter pylori* infection.

The diagnosis of EoE was established based on clinical presentation, histological findings, and exclusion of other identifiable causes of esophageal eosinophilia, including gastroesophageal reflux disease, drug-induced and infectious esophagitis, and Crohn's disease.

Pulmonary function testing showed a normal forced vital capacity (FVC) of 2.7 L (Z-score +0.9), a baseline forced expiratory volume in one second (FEV1) of 1.73 L (approximately 70% predicted), a reduced FEV1/FVC ratio of 64%, and a post-bronchodilator FEV1 of 2.21 L (approximately 89% predicted) after administration of 400 μg of salbutamol, corresponding to a significant reversibility of +28%.

Allergy assessment included skin-prick testing and serum-specific IgE measurement. Skin-prick testing was negative, and no sensitization to aeroallergens was detected. Food-specific IgE testing showed mild sensitization to prawns (0.66 IU/mL) and positivity to cross-reactive carbohydrate determinants (CCD) (2.0 IU/mL), while the remaining tested food allergens were negative. Total serum IgE levels were not available. In the absence of a clinical history suggestive of an immediate allergic reaction to prawns, these findings were interpreted as sensitization rather than evidence of clinically relevant food allergy. The positive CCD result may reflect cross-reactivity and may partly account for the detected prawn-specific IgE. Component-resolved diagnostics were not performed.

Treatment was initiated with swallowed topical corticosteroids (fluticasone 250 μg twice daily, delivered via a metered-dose inhaler and swallowed rather than inhaled, with instructions to avoid food and drink for 30 minutes afterward), combined with high-dose proton pump inhibitors (omeprazole 40 mg twice daily) and a six-food elimination diet (milk, egg, wheat, soy, peanuts, and fish), supervised by a pediatric dietitian.

The patient demonstrated rapid clinical improvement, with complete resolution of hematemesis and progressive weight gain. Follow-up endoscopic biopsies performed at 10 months showed histological remission, with fewer than 15 eosinophils per HPF and only mild nonspecific reactive changes.

The patient's clinical, endoscopic, histological, therapeutic, and outcome findings are summarized in Table 1.

**Table 1:** Summary of clinical, endoscopic, histological, therapeutic, and outcome findings. HPF; high-power field; PPI; proton pump inhibitors

Parameter	Finding
Age	13 years
Sex	Female
Respiratory history	Moderate persistent asthma
Gastrointestinal history	Previous *Helicobacter pylori* gastritis
Main presentation	Moderate hematemesis
Chronic symptom	Recurrent vomiting for one year
Admission signs	Tachycardia, hypoxemia
Hemoglobin	13.3 g/dL
Blood transfusion	Not required
Endoscopic rings	Present
White mucosal exudates	Present
Esophageal eosinophilia	>15 eosinophils/HPF
Food sensitization	Prawns
Topical corticosteroid	Swallowed fluticasone
Acid suppression therapy	High-dose PPI
Dietary therapy	Six-food elimination diet
Clinical outcome	Remission
Histological outcome	Remission at 10 months

## Discussion

EoE is a chronic inflammatory condition of the esophagus, defined by mucosal infiltration of at least 15 eosinophils per HPF on biopsy, in the absence of other identifiable causes [2,5].

Its pathophysiology involves a Th2-type immune response mediated by pro-inflammatory cytokines such as IL-5, IL-13, and eotaxin-3, which promote the recruitment, survival, and activation of eosinophils in the esophageal mucosa [3]. This inflammatory cascade is triggered by food or environmental antigens in genetically predisposed individuals [6].

EoE most commonly occurs in an atopic context, in association with asthma, allergic rhinitis, or eczema, reflecting a shared immune predisposition [3,4].

Our case concerns a 13-year-old female patient, an age consistent with pediatric literature data, which place the mean age at diagnosis between eight and 12 years, with a peak incidence among school-age children and adolescents [2,9].

Our patient had two notable medical histories: moderate persistent asthma and *Helicobacter pylori* gastritis. The association between EoE and asthma is well established in the literature, with the prevalence of asthma estimated at between 30% and 50% in children with EoE [3,4]. In their meta-analysis, González-Cervera et al. confirmed this significant association between atopic conditions and EoE, with asthma being the most commonly observed comorbidity [4].

As for *Helicobacter pylori*, its role in relation to EoE is more complex. Several studies have highlighted an inverse association between the prevalence of *Helicobacter pylori* and that of atopic diseases, including EoE. *Helicobacter pylori* has been proposed to promote regulatory T-cell responses, potentially attenuating Th2-mediated inflammation. However, the evidence remains inconclusive [10].

The clinical presentation of EoE is varied and closely linked to the patient's age. In infants and young children, the most common symptoms are vomiting, regurgitation, and abdominal pain, often mistaken for gastroesophageal reflux. In school-age children and adolescents, symptoms are predominantly dysphagia, food impaction, and retrosternal pain [1,5,9].

These symptoms are often intermittent and may delay diagnosis by several years; the average delay between symptom onset and diagnosis is estimated to be between two and six years [1,9].

In our case, this delay was limited to one year due to the sudden and alarming nature of the hematemesis, which prompted rapid investigation. While the initial presentation of recurrent vomiting is consistent with the literature, the occurrence of sudden-onset hematemesis of moderate abundance represents an atypical, though increasingly recognized, presentation of EoE.

Aydin and Beser reported the case of a 16-year-old girl admitted with profuse hematemesis, in whom endoscopy excluded a surgical cause and esophageal biopsies confirmed the diagnosis of EoE [7]. Similarly, Ozdogan et al. described the first pediatric case series presenting with upper gastrointestinal bleeding: five children aged seven to 12 years admitted for severe hematemesis, most of whom had an atopic background, peripheral hypereosinophilia, and elevated total IgE levels; histology confirmed eosinophilic infiltration ≥15/HPF with eosinophilic microabscesses [8]. Liacouras et al., in a large pediatric series, also reported upper gastrointestinal bleeding as a possible complication of severe active EoE [11].

Our case adds to these observations and highlights the need to include EoE in the differential diagnosis of upper gastrointestinal bleeding in children or adolescents with atopic conditions, even in the absence of prior dysphagia. Compared with previously reported cases, this observation is notable for the co-occurrence of an acute asthma exacerbation with the onset of hematemesis, a combination not previously emphasized in the pediatric EoE literature.

Emergency EGD revealed endoscopic findings suggestive of EoE, and multiple biopsies confirmed mucosal eosinophilic infiltration with more than 15 eosinophils per HPF and eosinophilic microabscesses, consistent with current diagnostic criteria [1,6]. Current guidelines recommend obtaining at least six biopsies from the proximal, middle, and distal esophagus due to the patchy nature of the disease [1], which was the approach followed in the present case.

From an allergological perspective, the findings suggest food sensitization to prawns, with no evidence of aeroallergen sensitization. This is consistent with the literature, which highlights the predominant role of food allergens in EoE, whereas aeroallergens appear to be implicated in a minority of cases [1,4]. However, positive specific IgE alone does not necessarily indicate a clinically relevant food allergy. In this case, the absence of a reported clinical reaction to prawn ingestion, together with the presence of CCD reactivity, makes the clinical significance of the prawn sensitization uncertain. The CCD positivity may reflect cross-reactivity rather than true allergen-specific sensitization. Therefore, the contribution of prawn sensitization to the patient's EoE remains uncertain based on the available data.

In accordance with current recommendations, a six-food elimination diet targeting milk, eggs, wheat, soy, peanuts, and fish was introduced. This strategy, first described by Kagalwalla et al., achieves histological remission in approximately 70% of pediatric cases without prior allergy testing [12].

Our patient's management was based on topical corticosteroids (fluticasone 250 μg twice daily), which represent the standard pharmacological treatment for EoE. These agents act locally on the esophageal mucosa, reducing eosinophilic inflammation, with reported histological remission rates ranging from 60% to 80% [1,13]. Proton pump inhibitors were maintained at high doses as adjunct therapy, given their dual role in controlling acid exposure and exerting anti-inflammatory effects through inhibition of eotaxin-3 and restoration of epithelial barrier function [1,6].

This combined therapeutic approach led to rapid clinical improvement, with resolution of symptoms, histological remission (<15 eosinophils/HPF), and progressive weight gain (+2 kg) by the 10-month follow-up, consistent with previously reported outcomes.

Alternative therapeutic options include viscous budesonide formulations, which provide prolonged mucosal contact [13], and dupilumab, a biologic therapy targeting the IL-4/IL-13 pathway for selected patients with treatment-resistant disease [14,15]. Finally, endoscopic dilation remains indicated in fibrosing forms with persistent dysphagia despite well-managed medical treatment, as an adjunct to this and not as a substitute [1,16].

This report is subject to certain limitations inherent to its single-case design, which limits the generalizability of these findings; in addition, some clinical parameters, such as the precise volume of blood loss, could not be retrospectively quantified.

## Conclusions

EoE represents an important cause of both chronic and acute gastrointestinal symptoms in children. Hematemesis, although rare, may constitute an inaugural presentation in atopic pediatric patients and should therefore be considered in the etiological assessment of any upper gastrointestinal bleeding, even in the absence of dysphagia. Diagnosis relies on EGD with multiple staged biopsies, which remains the only method capable of confirming the characteristic eosinophilic mucosal infiltration.

Treatment should be individualized and may include dietary elimination, swallowed topical corticosteroids, proton pump inhibitors, or a combination of these approaches, depending on the patient's clinical profile. Finally, biologic therapies represent a major therapeutic advance for refractory forms.

## References

[1] Gupta M, Grinman M (2024). Diagnosis and management of eosinophilic esophagitis. CMAJ.

[2] Hahn JW, Lee K, Shin JI (2023). Global incidence and prevalence of eosinophilic esophagitis, 1976-2022: a systematic review and meta-analysis. Clin Gastroenterol Hepatol.

[3] Licari E, Borish LC, Ghosal S (2026). Prevalence and predictors of eosinophilic esophagitis (EoE) among children with severe asthma. J Allergy Clin Immunol Pract.

[4] González-Cervera J, Arias Á, Redondo-González O, Cano-Mollinedo MM, Terreehorst I, Lucendo AJ (2017). Association between atopic manifestations and eosinophilic esophagitis: a systematic review and meta-analysis. Ann Allergy Asthma Immunol.

[5] Papadopoulou A, Koletzko S, Heuschkel R (2014). Management guidelines of eosinophilic esophagitis in childhood. J Pediatr Gastroenterol Nutr.

[6] Societe Francaise d’Endoscopie Digestive (2026). L’Œsophagite à éosinophiles de l’enfant et de l’adolescent. https://www.sfed.org/article-abecedaire/loesophagite-a-eosinophiles-de-lenfant-et-de-ladolescent/.

[7] Aydin E, Beser OF (2017). Profuse upper gastrointestinal bleeding secondary to eosinophilic esophagitis. APSP J Case Rep.

[8] Ozdogan E, Caglayan LD, Mizikoglu O, Arikan C (2020). Upper gastrointestinal bleeding as the first presentation of eosinophilic gastrointestinal disease. JPGN Rep.

[9] Plate J, Söderbergh T, Bergqvist J, Lingblom C, Bergquist H, Larsson H (2025). Eosinophilic esophagitis prevalence, incidence, and presenting features: a 22-year population-based observational study from southwest Sweden. Dis Esophagus.

[10] Arnold IC, Dehzad N, Reuter S, Martin H, Becher B, Taube C, Müller A (2011). Helicobacter pylori infection prevents allergic asthma in mouse models through the induction of regulatory T cells. J Clin Invest.

[11] Liacouras CA, Furuta GT, Hirano I (2011). Eosinophilic esophagitis: updated consensus recommendations for children and adults. J Allergy Clin Immunol.

[12] Kagalwalla AF, Sentongo TA, Ritz S (2006). Effect of six-food elimination diet on clinical and histologic outcomes in eosinophilic esophagitis. Clin Gastroenterol Hepatol.

[13] Straumann A, Conus S, Degen L (2010). Budesonide is effective in adolescent and adult patients with active eosinophilic esophagitis. Gastroenterology.

[14] Dellon ES, Rothenberg ME, Collins MH (2022). Dupilumab in adults and adolescents with eosinophilic esophagitis. N Engl J Med.

[15] Walter S, Ho J, Alvarado R (2022). Mepolizumab decreases tissue eosinophils while increasing type-2 cytokines in eosinophilic chronic rhinosinusitis. Clin Exp Allergy.

[16] Schoepfer AM, Gonsalves N, Bussmann C, Conus S, Simon HU, Straumann A, Hirano I (2010). Esophageal dilation in eosinophilic esophagitis: effectiveness, safety, and impact on the underlying inflammation. Am J Gastroenterol.

